# Diagnosing Gestational Diabetes with a Probably Too Simplified Diagnostic Procedure Compared to International Criteria: The Indian Case Study

**DOI:** 10.3390/jcm11133745

**Published:** 2022-06-28

**Authors:** Francesco Corrado, Antonino Di Benedetto, Giacoma Di Vieste, Laura La Fauci, Canio Martinelli, Rosario D’Anna, Basilio Pintaudi

**Affiliations:** 1 Department of Human Pathology, University of Messina, 98122 Messina, Italy; francesco.corrado@unime.it (F.C.); lauralafauci@gmail.com (L.L.F.); caniomartinelli.md@gmail.com (C.M.); rdanna@unime.it (R.D.); 2 Department of Internal Medicine, Policlinico Martino, University of Messina, 98122 Messina, Italy; antonino.dibenedetto@unime.it; 3 Diabetes Unit, Cantù Hospital, 20081 Abbiategrasso, Italy; giacoma.divieste@asst-ovestmi.it; 4 Diabetes Unit, Niguarda Cà Granda Hospital, 20162 Milan, Italy

**Keywords:** gestational diabetes, screening risk factors, diagnostic criteria

## Abstract

Diagnostic procedures for the diagnosis of gestational diabetes mellitus (GDM) are not uniformly defined worldwide. We retrospectively applied two diagnostic procedures (i.e., the IADPSG and the Indian) to the same pregnant women in order to compare the clinical characteristics and the prevalence of risk factors for GDM. Overall, 1015 pregnant women were evaluated. GDM was diagnosed in 113 cases (11.1%) by the IADPSG criteria and in 105 cases (10.3%) by the Indian criteria. The women diagnosed with GDM according to the IADPSG criteria had higher pre-gestational BMIs, higher previous macrosomia rates, higher first trimester fasting blood glucose levels, higher fasting and 1 h glucose levels after glucose load at OGTT, and lower 2 h glucose levels at OGTT compared with the women with GDM diagnosed according to the Indian criteria. Only 49.6% of the women who were diagnosed by the IADPSG criteria were also diagnosed with GDM by the Indian diagnostic criteria. For 47.8% of the women who were diagnosed by the IADPSG criteria, a diagnosis of GDM was missed by applying the Indian diagnostic criteria. Interestingly, 49 women were diagnosed with GDM by the Indian criteria but were normal according to the IADPSG criteria. Different diagnostic criteria could lead to different GDM detection rates with different practical approaches.

## 1. Introduction

Gestational diabetes mellitus (GDM) is defined as any degree of glucose intolerance that occurs for the first time or is first detected during pregnancy [1].

A general agreement on the need to diagnose and therefore successfully manage this condition in order to reduce the risk of maternal, fetal, and neonatal complications has been reached [2,3]. However, the diagnostic criteria for GDM are very heterogeneous worldwide [4]. One-step or two-step diagnostic procedures with or without a risk-based approach are used.

The criteria suggested by the International Association of Diabetes and Pregnancy Study Groups (IADPSG) are the most commonly used [5,6]. This one-step approach consists of performing a 75 g oral glucose tolerance test (OGTT) at 24–28 weeks’ gestation with fasting 1 h and 2 h after load plasma glucose measurements are taken. A diagnosis of GDM is made when any of the following is met or exceeded: 92 mg/dL (5.1 mmol/L) for fasting, 180 mg/dL (10.0 mmol/L) 1 h after load, or153 mg/dL (8.5 mmol/L) 2 h after load.

As many countries have limited financial resources, other diagnostic strategies are considered equally acceptable. In most cases, pragmatic country-specific guidelines for GDM diagnosis have been defined on the basis of available resources [7].

Interestingly, the Diabetes in Pregnancy Study Group of India advocates for universal screening using a single non-fasting 2 h 75 g glucose challenge test, with a 2 h value ≥ 140 mg/dL being diagnostic of GDM [8]. Our clinics are increasingly populated by women with GDM from south Asia, particularly from India. These women do not always perceive GDM as a condition that requires appropriate medical checks and attention for cultural reasons. They are also used to following clearly different diagnostic procedures than those used in other countries.

The aim of our study was to retrospectively evaluate two diagnostic procedures (i.e., IADPSG and Indian) within the same pregnant population. We wanted to compare the clinical characteristics and the prevalence of risk factors for GDM in order to understand how important the clinical impact of the diagnostic test is and how the prevalence of risk factors could vary between the two samples of women diagnosed as having GDM. This could help find the most effective and cost-saving diagnostic strategy for GDM.

## 2. Methods

This was an observational, retrospective, single-center study that was approved by the Ethics Committee (protocol number 117/2012) of the University of Messina, Italy. All the participants gave informed consent. The involved subjects were 1015 pregnant women consecutively referred to the Clinic of Diabetes and Pregnancy of the University of Messina, Italy. The inclusion criteria were: age >18 years, single pregnancy. The exclusion criteria were: a previous diagnosis of overt diabetes (i.e., type 1 or type 2 diabetes), age <18 years, no informed consent. All the women underwent a 75 g 2 h oral glucose test (OGTT) between 24 and 28 weeks of gestation to check for GDM presence. The diagnosis of GDM was made according to the IADPSG recommendations [6]. A 75 g OGTT with plasma glucose measurements at fasting, 1 h, and 2 h after glucose load was performed. The diagnosis of GDM according to the IADPSG criteria was made when any of the following plasma glucose values were exceeded: ≥92 mg/dL (5.1 mmol/L) for fasting glucose, ≥180 mg/dL (10.0 mmol/L) 1 h after glucose load, and ≥153 mg/dL (8.5 mmol/L) 2 h after glucose load. The diagnosis of GDM according to the Indian criteria was instead made if the glucose levels were ≥ 140 mg/dL (7.8 mmol/L) 2 h after glucose load [8]. The following information was collected from electronic clinical records: maternal age, pre-pregnancy BMI, parity, firsttrimester fasting plasma glucose (FPG) values, FPG values between 100 mg/dL (5.6 mmol/mol) and 125 mg/dL (6.9 mmol/L) during the pre-pregnancy period or in the first trimester of the pregnancy, previous GDM, previous macrosomia (i.e., birth weight ≥4500 g), family history of diabetes (i.e., first-degree relative with diabetes), and OGTT glucose values.

### Statistical Analysis

Data are reported as means and standard deviations for continuous variables, and percentages for categorical variables. First, a comparison between women with or without GDM according to the Indian criteria was performed. Second, the clinical characteristics of the women diagnosed with GDM by the IADPSG and Indian criteria were compared. The characteristics of the study population were also categorized into four groups according to the combination of the IADPSG and Indian criteria for GDM diagnosis: IADPSG-GDM no and Indian-GDM no, IADPSG-GDM yes and Indian-GDM no, IADPSG-GDM no and Indian-GDM yes, and IADPSG-GDM yes and Indian-GDM yes.Comparisons were performed using parametric and non-parametric tests according to variable distribution. A pre-specified sample size calculation was not performed. We would like to retrospectively compare the two diagnostic criteria in a large sample of women screened for GDM in our clinic. No specific clinical (i.e., maternal, fetal, or neonatal) outcome was considered in the comparison as a main outcome. A *p* value <0.05 was considered statistically significant. The analyses were carried out using SPSS Statistics 17.0 computer software (SPSS, Inc., Chicago, IL, USA).

## 3. Results

Overall, 1015 pregnant women, of whom 811 were Italian, were evaluated. GDM was diagnosed in 113 cases (11.1%) by the IADPSG criteria and in 105 cases (10.3%) by the Indian criteria. The women diagnosed with GDM according to the Indian criteria (i.e., those with 2 h OGTT glucose ≥140 mg/dL, 7.8 mmol/L) were older, had higher pre-gestational BMIs, higher first trimester fasting blood glucose levels, higher fasting, 1 h, and 2 h glucose levels after glucose load at OGTT, and more often had a family history of diabetes and a history of previous GDM compared with the women with no GDM (i.e., those with 2 h OGTT glucose <140 mg/dL, 7.8 mmol/L) (data not shown).

The clinical characteristics of the study population diagnosed with GDM according to the IADPSG and Indian criteria are reported in Table 1. The women diagnosed with GDM according to the IADPSG criteria had higher pre-gestational BMIs, higher previous macrosomia rates, higher first trimester fasting blood glucose levels, higher fasting and 1 h glucose levels after glucose load at OGTT, and lower 2 h glucose levels at OGTT compared with the women with GDM diagnosed according to the Indian criteria.

The clinical characteristics of the study population according to the categorization of the study population into four groups are reported in Table 2.

Only 56 women, representing 49.6% of the women who were diagnosed by the IADPSG criteria, were diagnosed with GDM when applying both the IADPSG and the Indian diagnostic criteria. Therefore, for these women, there was an overlapping diagnosis. For 54 women, representing 47.8% of the women who were diagnosed by the IADPSG criteria, a diagnosis of GDM was missed when applying the Indian diagnostic criteria. This means that the diagnosis was made according to their impaired fasting or 1 h OGTT glucose levels. Interestingly, 49 women were diagnosed with GDM by the Indian criteria but were normal according to the IADPSG criteria, meaning that their fasting and 1 h OGTT glucose levels were normal and their 2 h OGTT glucose levels were ≥140 mg/dL (7.8 mmol/L) but <153 mg/dL (8.5 mmol/L).

## 4. Discussion

Our study compared two diagnostic procedures for the identification of quite a frequent condition, gestational diabetes. We reported a different prevalence of GDM resulting from the application of the strategy proposed by the IADPSG and the diagnostic criteria used in India. The women diagnosed with GDM by the IADPSG criteria had a higher prevalence of classic risk factors for GDM compared with the women with GDM identified according to the Indian criteria. For example, they had higher pre-gestational BMI and higher first trimester fasting blood glucose levels, two well-known independent risk factors for the onset of GDM and for the occurrence of neonatal complications. We detected a very high percentage of the women (47.8%) who were diagnosed with GDM by the IADPSG criteria but whose diagnosis of GDM was missed when applying the Indian diagnostic criteria. This suggests that the two different diagnostic strategies are not comparable and identify different cohorts of women.

The choice of the best diagnostic test and the most suitable screening procedure for GDM still represents a problem. In fact, many health organizations worldwide have not yet defined which are the most clinically useful and cost-effective procedures for testing women for GDM. This issue not only involves the most industrialized and civilized countries, which are able to offer high-level analytical and diagnostic methods, but also involves countries with sometimes extremely limited economic resources. This hinders the feasibility of diagnostic tests conducted under standardized conditions. This important aspect of global health policy was highlighted a few years ago by the FIGO (International Federation of Gynecology and Obstetrics), which highlighted how the possibility of diversifying diagnostic procedures exists in countries with scarce or very scarce health resources [7]. An example of these countries is India. In this country, in fact, there is an objective challenge for diagnostic centers represented by hospitals or some health facilities in which screening tests are periodically conducted. The women to be tested for GDM have to go to the centers starting from places of residence that are far from the centers themselves. This implies the difficulty of performing basal sampling in optimal conditions. In fact, women are unable to arrive in a fasting condition as they are unable to arrive at the diagnostic centers early in the morning to perform basic tests. Furthermore, due to the effects of physiological hormones with counterinsular action, the reliability of the blood glucose values found in a so-called basal sample is strongly conditioned, hence the need to remedy this lack of reliability of the basal values by essentially exploiting the post-load glucose values. As there is no international gold standard for comparing diagnostic methods, our study had the prerogative of comparing the diagnostic test used in India with the most widespread internationally, represented by that proposed by the IADPSG. Therefore, it is interesting to compare diagnostic methods that include glucose basal values with tests performed only with post-load values. Clustering the samples according to different diagnostic criteria highlighted how the combination of different risk factors could impact the onset of GDM. The results of our study are in line with those reported by a systematic review and meta-analysis on the screening and diagnosis of GDM in India [9]. The authors showed that the IADPSG diagnostic criteria found significantly more GDM cases than the WHO 1999 criteria (prevalence 19.2% vs. 10.1%, respectively) and Indian criteria (7.4%). GDM prevalence is different across the country because of geography and population types. This may explain some of the difficulty involved in establishing a national program for GDM diagnosis. Genetics and different cultures could explain the higher prevalence rates of GDM in urban populations.

A recent publication compared the diagnostic accuracy of the Indian criteria with the Carpenter and Coustan and National Diabetes Data Group (NDDG) criteria for the diagnosis of GDM [10]. The authors also tested eventual correlation with fetal–maternal outcomes. The study concluded that the diagnostic accuracy, sensitivity, and specificity of the Indian criteria are comparable to those of the Carpenter and Coustan and NDDG criteria. For these reasons, the Indian criteria were recommended for diagnosing GDM with the added advantages of low cost, simplicity, and convenience. Our study aimed to compare the Indian criteria with the IADPSG criteria. The latter are different from the Carpenter and Coustan and NDDG criteria and have more strength in terms of associations with maternal and neonatal outcomes. A direct overlap between the various criteria is therefore not possible.

The choice of the best screening and diagnostic procedures is also a problem for health care professionals. With the aim of exploring the perspectives of healthcare providers regarding the barriers from the health system context that restrict the timely screening and effective management of GDM, a qualitative study was performed [11].

The barriers to GDM screening included delayed visits to public hospitals and the stress of household-level responsibilities. The migration of pregnant women to their natal homes during their first pregnancy represents a cultural barrier. Other recognized barriers were related to the national health system: long waiting hours, lack of follow-up, resource scarcity, and lack of supportive oversight.

Our study underlines important implications for clinical practice. First, because it highlights that different sub-categories of women could be negative for GDM according to the international criteria but positive according to the Indian criteria, there is a need for further studies aimed at understanding what the most important risk factors are and what combinations of risk factors are most capable of determining adverse neonatal outcomes independently of predefined screening and diagnosis strategies.

Some scientific research has focused on the diagnosis and treatment of particular subgroups of women with a high risk of developing GDM. These groups of women have been determined by screening and diagnosis algorithms that are based on some clinical parameters readily available on the occasion of outpatient visits [12,13]. Our study showed that an accurate assessment of the antenatal risk factors for GDM is needed. This evaluation allows for the better identification of women at high risk of the development of GDM compared with a simplified diagnostic procedure such as the Indian one.

Our study certainly has some limitations typical of observational studies. Its most important limitation is the lack of maternal and neonatal outcomes. This did not allow us to detect potential associations between the diagnostic test for GDM and the critical pregnancy outcomes.

## 5. Conclusions

The oral glucose tolerance test is not always accurate for GDM diagnosis due to its reproducibility and, in some cases, also due to the difficulty of correct execution. The Indian case is not the only example. Countries with limited resources should have a different GDM diagnostic strategy, independent of laboratory tests. A clinical score or algorithm capable of detecting women at higher risk of developing neonatal complications could be useful in clinical practice. In addition, health care professionals should be careful when caring for women with GDM diagnosed in countries with different diagnostic criteria or with a diagnosis made with methods that are not fully reliable.

## Figures and Tables

**Table 1:** Clinical characteristics of women with GDM diagnosed by IADPSG and Indian criteria.

	IADPSG	Indian	*p*	95%CI
N (%)	113 (11.1)	105 (10.3)		
Age (years)	32.0 ± 4.8	32.2 ± 4.9	0.78	0.94–1.08
Age ≥35 years (%)	31.0	37.1	0.23	0.31–1.33
Pre-gestational weight (kg)	67.3 ± 13.6	64.5 ± 13.9	0.005	0.56–1.27
Pre-gestational BMI (kg/m^2^)	25.7 ± 4.7	24.4 ± 4.8	<0.0001	0.59–1.36
Pre-gestational BMI ≥25 kg/m^2^ (%)	49.0	40.0	0.71	0.58–2.24
Women at first pregnancy (%)	56.6	57.1	0.79	0.56–2.14
Family history of diabetes (%)	40.7	41.9	0.75	0.56–2.21
Previous GDM (%)	8.8	7.6	0.38	0.10–2.42
Previous macrosomia (%)	6.2	0.0	<0.0001	-
First trimester fasting blood glucose (mg/dL)	87.1 ± 10.3	82.9 ± 10.6	<0.0001	−0.02–−0.004
First trimester fasting blood glucose between 100 and 125 mg/dL (%)	10.6	7.6	0.96	0.29–3.60
Fasting glucose at OGTT (mg/dL)	88.1 ± 9.0	82.3 ± 9.5	0.003	−0.03–−0.01
1 h after glucose load at OGTT (mg/dL)	175.9 ± 28.2	169.4 ± 27.8	<0.0001	0.20–1.10
2 h after glucose load at OGTT (mg/dL)	140.9 ± 25.8	155.5 ± 12.3	<0.0001	0.01–0.02

**Table 2:** Clinical characteristics of the study population according to the combination of IADPSG and Indian diagnostic criteria for gestational diabetes.

	IADPSG-GDM No and Indian-GDM No	IADPSG-GDM Yes and Indian-GDM No	IADPSG-GDM No and Indian-GDM Yes	IADPSG-GDM Yes and Indian-GDM Yes
N (%)	839 (83.8)	54 (5.4)	49 (4.9)	56 (5.6)
Age (years)	30.5 ± 5.4	31.9 ± 4.6	32.2 ± 4.7	32.1 ± 5.1
Age ≥35 years (%)	22.5	25.9	40.8	33.9
Pre-gestational weight (kg)	61.3 ± 11.3	68.2 ± 14.1	61.6 ± 14.6	66.9 ± 12.9
Height (cm)	161.5 ± 9.7	162.4 ± 6.8	162.5 ± 6.3	162.5 ± 5.8
Pre-gestational BMI (kg/m^2^)	23.8 ± 6.9	25.8 ± 4.9	23.2 ± 4.9	25.4 ± 4.6
Pre-gestational BMI ≥25 kg/m^2^ (%)	26.7	46.3	28.6	50.0
Women at first pregnancy (%)	52.1	51.9	53.1	60.7
Family history of diabetes (%)	21.1	42.6	40.8	42.9
Previous GDM (%)	0.0	3.7	0.0	14.3
Previous macrosomia (%)	1.3	13.0	0.0	0.0
First trimester fasting blood glucose (mg/dL)	77.9 ± 7.7	88.3 ± 9.0	80.0 ± 9.0	85.4 ± 11.4
First trimester fasting blood glucose between 100 and 125 mg/dL (%)	0.1	7.4	0.0	14.3
Fasting glucose at OGTT (mg/dL)	77.7 ± 6.3	90.0 ± 7.6	78.0 ± 7.3	86.1 ± 9.6
1 h after glucose load at OGTT (mg/dL)	120.1 ± 24.1	169.4 ± 30.6	154.7 ± 25.1	182.3 ± 23.5
2 h after glucose load at OGTT (mg/dL)	102.0 ± 18.8	120.0 ± 16.8	149.3 ± 7.0	160.9 ± 13.3

## Data Availability

Data are available from the corresponding author upon reasonable request.

## Institutional Review and Board Statement

The study was conducted according to the guidelines of the declaration of Helsinki and approved by the Ethics Committee of the University of Messina.

## References

[1] Metzger B.E., Coustan D.R., Committee O. (1998). Summary and recommendations of The Fourth International workshop-conference on gestational diabetes mellitus. Diabetes Care.

[2] Crowther C.A., Hiller J.E., Moss J.R., McPhee A.J., Jeffries W.S., Robinson J.S. (2005). Australian Carbohydrate Intolerance Study in Pregnant Women (ACHOIS) Trial Group. Effect of treatment of gestational diabetes mellitus on pregnancy outcomes. N. Engl. J. Med..

[3] Landon M.B., Spong C.Y., Thom E., Carpenter M.W., Ramin S.M., Casey B., Wapner R.J., Varner M.W., Rouse D.J., Thorp J.M. (2009). A multicenter, randomized trial of treatment for mild gestational diabetes. N. Engl. J. Med..

[4] RamezaniTehrani F., Naz M.S.G., Yarandi R.B., Behboudi-Gandevani S. (2021). The Impact of Diagnostic Criteria for Gestational Diabetes Mellitus on Adverse Maternal Outcomes: A Systematic Review and Meta-Analysis. J. Clin. Med..

[5] Metzger B.E., Lowe L.P., Dyer A.R., Trimble E.R., Chaovariner U., Coustan D.R., HAPO Study Cooperative Research Group (2008). Hyperglycemia and adverse pregnancy outcomes. N. Engl. J. Med..

[6] Metzger B.E., Gabbe S.G., Persson B., Buchanan T.A., Catalano P.A., Damm P., Dyer A.R., Leiva A.D., Hod M., Kitzmiler J.L. (2010). International Association of Diabetes and Pregnancy Study Groups recommendations on the diagnosis and classification of hyperglycemia in pregnancy. Diabetes Care.

[7] McIntyre H.D., Colagiuri S., Roglic G., Hod M. (2015). Diagnosis of GDM: A suggested consensus. Best Pract. Res. Clin. Obstet. Gynaecol..

[8] Seshiah V. (2010). Diabetes in pregnancy study G. Fifth national conference of diabetes in pregnancy study group, India. J. Assoc. Physicians India.

[9] Li K.T., Naik S., Alexander M., Mathad J.S. (2018). Screening and diagnosis of gestational diabetes in India: A systematic review and meta-analysis. Acta Diabetol..

[10] Saxena P., Shubham T., Puri M., Jain A. (2022). Diagnostic Accuracy of Diabetes in Pregnancy Study Group of India with Carpenter-Coustan and National Diabetes Data Group Criteria for Diagnosis of Gestational Diabetes Mellitus and Correlation with Fetomaternal Outcome. J. Obstet. Gynaecol. India.

[11] Sahu B., Babu G.R., Gurav K.S., Karthik M., Ravi D., Lobo E., John D.A., Oakley L., Oteng-Ntim E., Nadal I.P. (2021). Health care professionals’ perspectives on screening and management of gestational diabetes mellitus in public hospitals of South India—A qualitative study. BMC Health Serv. Res..

[12] Pintaudi B., Fresa R., Dalfrà M., Dodesini A.R., Vitacolonna E., Tumminia A., Sciacca L., Lencioni C., Marcone T., Lucisano G. (2018). The risk stratification of adverse neonatal outcomes in women with gestational diabetes (STRONG) study. Acta Diabetol..

[13] Kuo C.H., Li H.Y. (2019). Diagnostic Strategies for Gestational Diabetes Mellitus: Review of Current Evidence. Curr. Diabetes Rep..

